# Niche partitioning in a sympatric cryptic species complex

**DOI:** 10.1002/ece3.1965

**Published:** 2016-01-28

**Authors:** Jessica J. Scriven, Penelope R. Whitehorn, Dave Goulson, Matthew. C. Tinsley

**Affiliations:** ^1^ Biological and Environmental SciencesSchool of Natural SciencesUniversity of StirlingStirlingFK9 4LAUK; ^2^ School of Life SciencesUniversity of SussexFalmerBrightonBN1 9QGUK

**Keywords:** *Bombus*, coexistence, community, diet, ecological divergence, niche overlap, niche region, PCR‐RFLP, pollinators, specialisation

## Abstract

Competition theory states that multiple species should not be able to occupy the same niche indefinitely. Morphologically, similar species are expected to be ecologically alike and exhibit little niche differentiation, which makes it difficult to explain the co‐occurrence of cryptic species. Here, we investigated interspecific niche differentiation within a complex of cryptic bumblebee species that co‐occur extensively in the United Kingdom. We compared the interspecific variation along different niche dimensions, to determine how they partition a niche to avoid competitive exclusion. We studied the species *B. cryptarum*,* B. lucorum,* and *B. magnus* at a single location in the northwest of Scotland throughout the flight season. Using mitochondrial DNA for species identification, we investigated differences in phenology, response to weather variables and forage use. We also estimated niche region and niche overlap between different castes of the three species. Our results show varying levels of niche partitioning between the bumblebee species along three niche dimensions. The species had contrasting phenologies: The phenology of *B. magnus* was delayed relative to the other two species, while *B. cryptarum* had a relatively extended phenology, with workers and males more common than *B. lucorum* early and late in the season. We found divergent thermal specialisation: In contrast to *B. cryptarum* and *B. magnus*,* B. lucorum* worker activity was skewed toward warmer, sunnier conditions, leading to interspecific temporal variation. Furthermore, the three species differentially exploited the available forage plants: In particular, unlike the other two species, *B. magnus* fed predominantly on species of heather. The results suggest that ecological divergence in different niche dimensions and spatio‐temporal heterogeneity in the environment may contribute to the persistence of cryptic species in sympatry. Furthermore, our study suggests that cryptic species provide distinct and unique ecosystem services, demonstrating that morphological similarity does not necessarily equate to ecological equivalence.

## Introduction

According to competition theory, ecologically similar coexisting species must partition resources (Hardin 1960). Multiple species should not be able to occupy the same niche indefinitely, as the best adapted species should eventually exclude inferior species from a given location (Gause 1932; Holt et al. 1994). Closely related species are often morphologically, physiologically, and behaviorally similar. Morphologically, similar species are expected to be ecologically alike and exhibit little niche differentiation (Violle et al. 2011; Cothran et al. 2013). This makes it difficult to explain the co‐occurrence of cryptic species, which are distinct species with similar or identical morphology, often historically “hidden” under a single species name and thus wrongly classified (Bickford et al. 2007; Williams et al. 2012b). Indeed, some studies have found that cryptic species are less likely to co‐occur than congeneric noncryptic species (Vodă et al. 2015a,b). Yet, cryptic species are common in nature and often do co‐occur at local scales (Ortells et al. 2003; Feulner et al. 2006; Stuart et al. 2006; Gabaldón et al. 2013; Van Campenhout et al. 2014); this makes them important test cases for studying the mechanisms that facilitate species coexistence. In this study, we investigated interspecific niche differentiation within a bumblebee cryptic species complex, comparing the degree to which species vary in different niche dimensions.

Approximately 250 species of bumblebees exist worldwide, distributed across the temperate, alpine, and arctic regions of the Northern Hemisphere and also South America. In much of this range, it is common for multiple species to occur in sympatry despite high niche overlap. Morphologically, most bumblebee species are very similar, with obvious differences only in size, tongue length, and coloration (Goulson and Darvill 2004; Goulson 2010). As they also all rely exclusively on pollen and nectar for food, theory would predict that bumblebee communities should be shaped by high levels of interspecific competition for these resources (Heinrich 1976; Inouye 1978).

**Figure 1:**
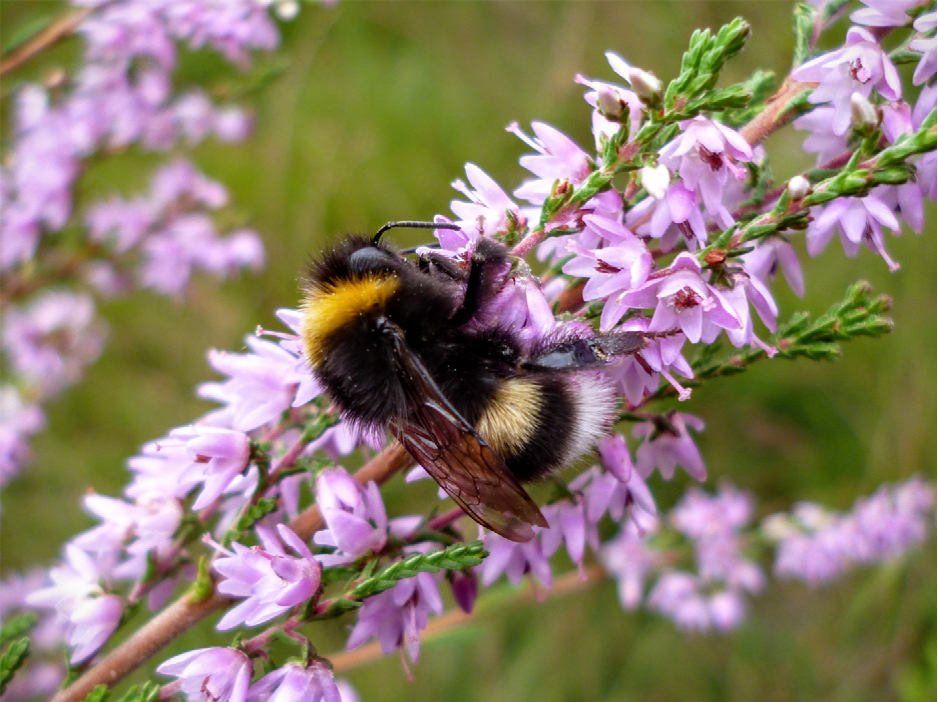
One of the *lucorum* complex species, which are morphologically indistinguishable in the field. This individual was feeding on heather in Glencoe. Photograph credit: Jessica Scriven.

*Bombus* species are notorious for possessing convergent color patterns between species, but also for displaying high intraspecific variation (Ellis et al. 2006; Williams 2007; Williams et al. 2012a). The subgenus *Bombus sensu stricto* is a widespread and commercially exploited taxon of bumblebee, comprising 17 species worldwide (Williams et al. 2012b), of which five are found in Europe: *Bombus* (*Bombus*) *cryptarum,* (Fabricius), *B*. (*B*.) *lucorum* (Linnaeus), *B*. (*B*.) *magnus* (Vogt), *B*. (*B*.) *sporadicus* (Nylander), and *B. (B.) terrestris* (Linnaeus). The taxonomic status of the latter two species is well established, but *B. lucorum*,* B. magnus* and *B. cryptarum* are morphologically indistinguishable in much of their range (Fig. 1), which has triggered considerable debate about their species status. *B. magnus* and *B. cryptarum* have previously been regarded by some as subspecies of *B. lucorum* and are often referred to collectively as the “*lucorum* complex,” or simply synonymized to *B. lucorum* (Benton 2006; Edwards & Jenner 2005). However, these three species are now recognised as a cryptic species complex: Studies on labial gland secretions have shown discrete genetic differences between the three species (Bertsch et al. 2005), as have studies of the CO1 gene (Carolan et al. 2012; Murray et al. 2007; Williams et al. 2012b), which suggest that *B. magnus* and *B. cryptarum* are more closely related to each other than to *B. lucorum* (Bertsch et al. 2005; Murray et al. 2008; Williams et al. 2012b). Morphological diagnostic characters have been proposed for queens, but some of these vary along a continuum, overlapping considerably between species, making them unreliable for identification (Carolan et al. 2012).

With a history of identification difficulties, relatively little is known about the field ecology of these cryptic *lucorum* complex species; in particular, the details of how they differentially exploit their general niche remain unclear. The three species are morphologically and ecologically similar; they are all short‐tongued species, meaning they have potential foraging access to the same floral resources. They occur sympatrically with broadly overlapping ranges in the UK and Ireland (Murray et al. 2007; Waters et al. 2011; Williams et al. 2012b; Stanley et al. 2013; Scriven et al. 2015). All three species co‐occur at many locations in Great Britain and Ireland; additionally, Scriven et al. (2015), Murray et al. (2007), and Stanley et al. (2013) found *B. lucorum* to be present at every site surveyed, suggesting that it is a relative ecological generalist. The only study in which *B. lucorum* was found to be absent from some locations was carried out in the far north‐west of Great Britain (Waters et al. 2011). Studies of geographic distributions have suggested that the *lucorum* complex species may be adapted to exploit different climatic conditions (Waters et al. 2011; Scriven et al. 2015): Unlike *B. lucorum*, both *B. cryptarum* and *B. magnus* occurred most commonly at sites with lower summer temperatures (Scriven et al. 2015). Furthermore, Scriven et al. (2015) and Waters et al. (2011) found that *B. magnus* was strongly associated with the forage plants *Calluna vulgaris* and *Erica* spp. and consequently with heathland habitats where these ericaceous plants were present.

Bumblebees and some other pollinators have recently suffered declines in abundance and range contractions across much of Western Europe and North America (Williams 1982, 2005; Goulson et al. 2008; Goulson 2010; Cameron et al. 2011). In the UK, seven of the 27 species are listed as priority species in the UK post‐2010 Biodiversity Framework (previously Biodiversity Action Plan), a higher proportion than for any other invertebrate group (Goulson 2010). Thus, as well as enabling us to test fundamental ecological theories, a thorough understanding of niche use in bumblebees has important conservation implications. This is especially critical for *B. magnus*, which is the rarest of the *lucorum* complex species and is tightly associated with threatened heathland habitat (Waters et al. 2011; Scriven et al. 2015).

In this study, we determine how these three cryptic species, *B. lucorum*,* B. cryptarum,* and *B. magnus*, partition a niche to avoid competitive exclusion. For the first time, we characterise the niches of these species at a single site across the duration of their flight season. We assess niche differentiation in three different ecological dimensions: Patterns of temporal activity, weather sensitivity, and forage resource use. In doing so, we aim to test which of these niche‐use phenotypes has most flexibly responded to the selection pressures generated by interspecific competition to facilitate niche differentiation and species co‐occurrence.

## Methods

### Sampling

The study site was the area in and around Glencoe village in the Highlands of Scotland, UK. A previous study found all three *lucorum* complex species in good numbers at this site (Scriven et al. 2015). Sampling was carried out below 150 m altitude within a 3 km radius of 56.68° N and ‐5.09° W, which included two villages and the bottom of Glencoe valley. The site was visited repeatedly between 30 April and 2 October 2014 on average every 11 days (interval: max. 13 days, min. 9 days). Sampling was carried out over approximately 2 days per visit (max. 3 days and min. 1 day). Road verges, paths, and any other accessible areas were searched continuously on foot throughout the day, from early morning until the evening; the exact times changed according to daylight hours throughout the season. Routes walked were varied so that all areas were visited at different times of day. Bumblebees resembling the *lucorum* complex species were caught and placed in a queen marking cage. For each individual captured, we recorded the following: date, time of day, forage plant, temperature (°C) using a TES Dual K‐type Thermometer (model 1312A), wind speed (m/sec) using an Airflow Developments anemometer (model LCA6000), amount of sun (scale 0–4, Table S1) and amount of rain (scale 0–5, Table S2). A single tarsus was removed from each individual and stored in absolute ethanol for subsequent DNA extraction, after which bees were released. All bees were checked for missing tarsi to prevent sampling the same individuals twice. Bees were predominantly captured when foraging on flowers. Early in the season queens were observed foraging high in the canopy of *Acer pseudoplatanus* and *Salix* sp. trees, it was not possible to catch these individuals; therefore, they are not included in this study.

### Species identification

DNA was extracted from tarsal samples using Chelex^®^ 100 (Walsh et al. 1991). For species identification, we used a PCR‐RFLP method, digesting an amplified fragment of the cytochrome oxidase I (COI) gene following Murray et al. (2008): This yields a diagnostic digestion pattern for each of the cryptic *lucorum* complex species and *B. terrestris*. Samples that did not produce unambiguous results after two attempts were discarded. Of the 519 bees sampled, 4.2% were identified as *B. terrestris*, some workers of which are morphologically similar to *B. lucorum* workers (Wolf et al. 2010). These *B. terrestris* individuals were excluded from further analyses.

### Analyses

All analyses were carried out using R version 3.0.2 (R Core Team 2014). To reduce the number of weather‐related explanatory variables, we employed principal component analysis (PCA) using the FactoMineR package (ver. 1.28, Lê et al. 2008). All variables were scaled to unit variance prior to analysis. PCA scores for axis 1 were associated with the level of sunshine and temperature (Figure S4B); this PCA variable (PCA 1) was used in some subsequent analyses.

To compare the seasonal and daily activity of the three bumblebee species and determine whether they were differentially affected by weather variables, we performed pairwise analyses between the species. We used generalised linear models with binary error distributions to test the association between these variables and the relative probability a sampled bumblebee belonged to a particular species within each pair. This analysis was performed separately for each caste (overwintered queens, workers, males); *B. magnus* was excluded from the analysis of males due to low sample size. Optimal models were selected to minimise AICc using the function dredge in the MuMIn package (ver. 1.9.5; Barton 2013) to run a complete set of models with all combinations of fixed effects and their two‐way interactions.

To define the niche of each species, we used the nicheROVER package (ver. 1.0; Swanson et al. 2015); we calculated the niche region (N_R_) for each of the three bumblebee species and the degree of niche overlap, based on phenology, time of day and sensitivity to weather variables. N_R_ is defined as a specific region of parameter space in which a randomly chosen individual has the probability *α* of being found (Swanson et al. 2015). For these analyses, *α *= 95%. Niche overlap was calculated as the probability that a sampled individual from species *A* was found in the N_R_ of species *B* (Swanson et al. 2015). Analyses were carried out separately for queens and workers; sample sizes for males were too small.

We determined diet differences between bumblebee species in three separate comparisons: for queens, workers, and males. We took the records of the flower species that each captured bee was visiting and used rarefaction to account for differences in sample size in our calculations of diet breadth (Williams et al. 2007). We noted the number of observations for the bee species with the fewest samples, rounded this down to the nearest multiple of five, and then drew this number of random samples of foraging observations without replacement for each bumblebee species. We drew 100 replicate subsamples per bee species to estimate the mean number of plant taxa each bee species would be expected to visit during a comparable number of flower visits, then determined the number of forage plant species in these subsamples. The preference of each bumblebee species for the ericaceous plants *Calluna vulgaris* or *Erica* spp. was examined using generalised linear models with individual bee as the unit of replication. The binary response represented whether the bee was recorded foraging on a *C. vulgaris*/*Erica* spp. flower (1) or any other plant species (0) and bumblebee species was used as the explanatory variable. This analysis was performed separately for queens and workers. We also calculated dissimilarity between the diets of the three species as Bray–Curtis distance measures (Bray and Curtis 1957) employing the Vegan package (ver. 2.3‐0, Oksanen et al. 2015) using relative abundances.

## Results

### Species identification

Of the 497 bees that belonged to the *lucorum* complex, 51.7% were *B. cryptarum,* 40.4% were *B. lucorum*, and 7.8% were *B. magnus*. Queens of the three species were similarly abundant; however, in comparison with *B. lucorum* and *B. cryptarum*, workers and males of *B. magnus* were relatively rare (Table 1).

**Table 1:** The total number of queens, workers, and males sampled for each species

Species	Queens	Workers	Males	Total
*B. lucorum*	21	153	27	201
*B. cryptarum*	26	174	57	257
*B. magnus*	23	14	2	39
Total	70	341	86	497

### Interspecific differences in phenology and diurnal activity

**Figure 2:**
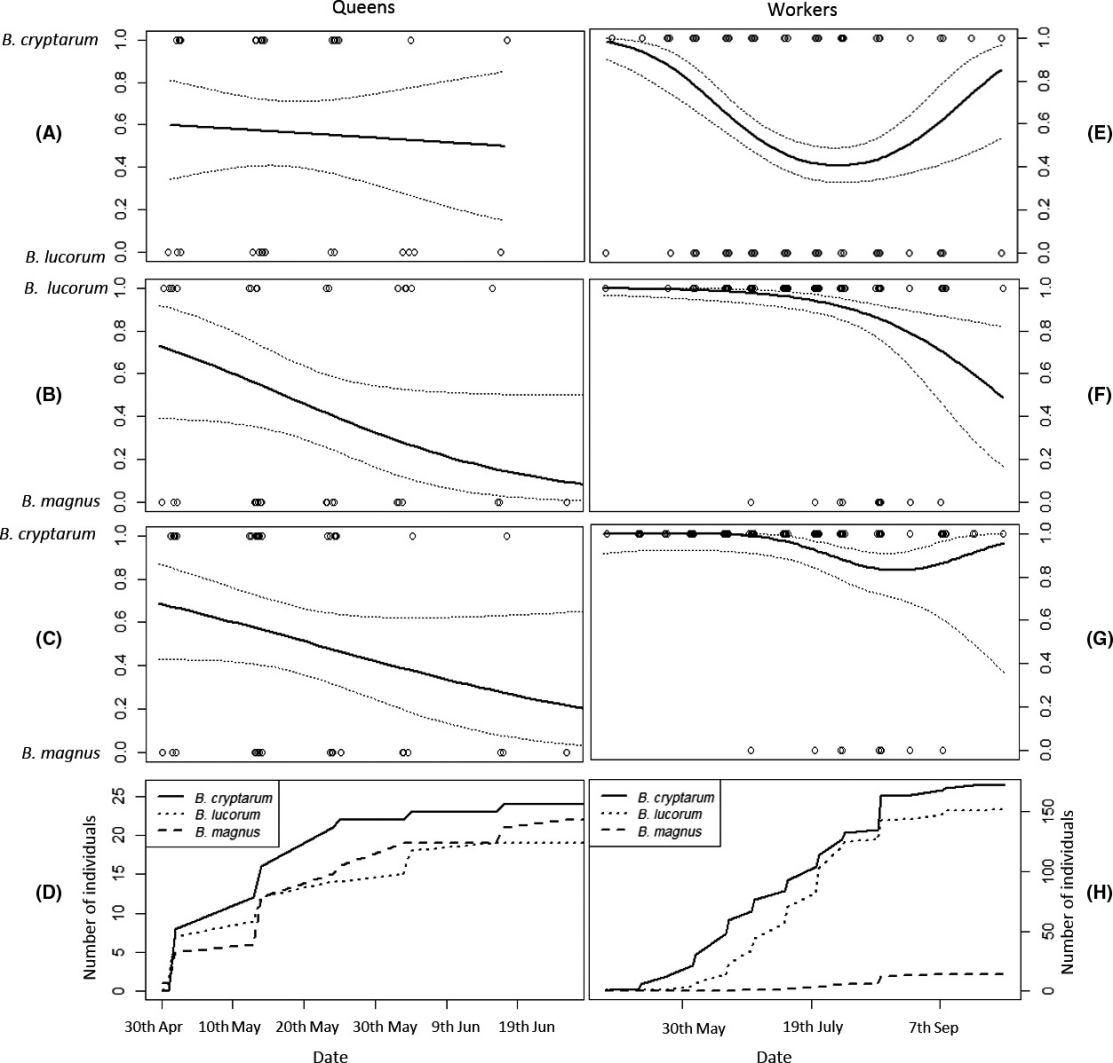
Interspecific variation in phenology of queens (A–D) and workers (E–H) of the *lucorum* complex species. Panels show the changes in the probability of an individuals belonging to the following: *B. cryptarum* compared to *B. lucorum* (A & E), *B. lucorum* compared to *B. magnus* (B & F), and *B. cryptarum* compared to *B. magnus* (C & G) as a function of date. The relative abundance of species pairs changed significantly through the season for all comparisons except for between *B. cryptarum* and *B. lucorum* queens (A; see S. 7). Trend lines are model fits from generalised linear models representing quadratic relationships in (E & G) and linear relationships in (B, C & F); 95% confidence intervals are shown around these relationships. Panels D & H show how the cumulative abundance of overwintered queens (D) and workers (H) shifted through the season for each bumblebee species.

We found interspecific phenological differences for each of the separate castes. For overwintered queens, *B. magnus* was scarcer than the other two species early in the season but became relatively more common as the season progressed (Fig. 2B–D & Table S3). In contrast, there was no significant difference in the dates when *B. cryptarum* and *B. lucorum* queens were on the wing (Fig. 2A,D & Table S3).

The relative abundance of foraging workers of the three species also varied throughout the season, reflecting distinct phenologies. Relative to either *B. lucorum* or *B. cryptarum*,* B. magnus* workers were significantly more common later in the season than they were at the beginning (Fig. 2F–H & Table S5). This meant that the period during which *B. magnus* workers were active coincided with the flowering of *Erica cinerea* and *C. vulgaris*. All *B. magnus* workers (*n* = 14) were on the wing when heather was flowering, whereas a lower percentage of all *B. cryptarum* (81%, *n* = 174) and *B. lucorum* (95%, *n* = 153) workers were flying at this time; nevertheless, this interspecific difference in the degree of activity bias toward the heather flowering season was not significant (Fisher Exact test *P* > 0.1). Comparing *B. cryptarum* and *B. lucorum* phenology, we found that at the beginning and end of the season, *B. cryptarum* workers were more common than *B. lucorum,* but in between, both species were equally abundant (Fig. 2E & Table S5). The strength of this seasonal shift in relative abundance varied according to the weather conditions (see below; Figs. S5 & S6).

**Figure 3:**
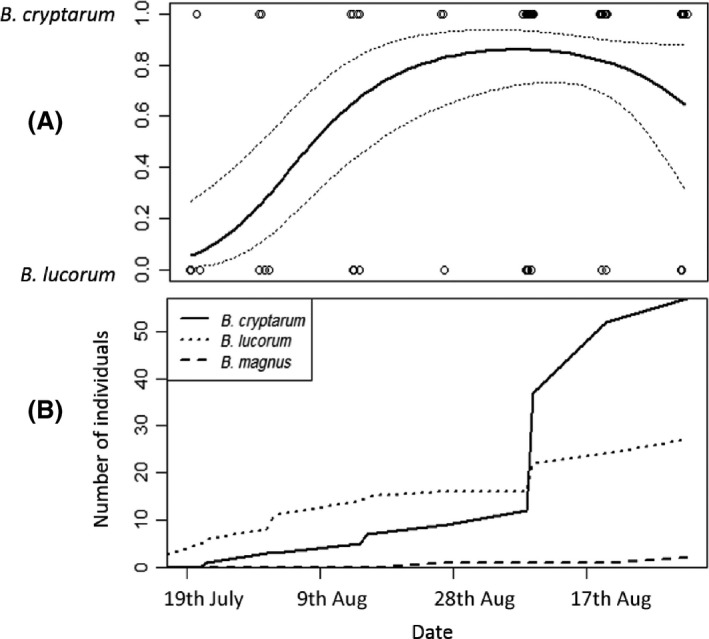
Interspecific variation in phenology of males of *B. cryptarum, B. lucorum* and *B. magnus*. (A) the probability that a male belonged to either *B. cryptarum* or *B. lucorum* changed significantly through the season. The trend line shows a quadratic relationship fit from a generalised linear model with 95% confidence intervals. The numbers of *B. magnus* males were too low to perform this analysis. (B) Changes in the cumulative number of males of each of the three bumblebee species over the season.

Considering new reproductives, when males were first encountered (21 July), they were mostly *B. lucorum* but this trend reversed significantly later in the season so that *B. cryptarum* males became more common (Fig. 3 & Table S7). Only two *B. magnus* males were found in the entire study, but the first of these was found over a month later (27 August) than when the first *B. cryptarum* and *B. lucorum* males appeared (Fig. 3B). Only five new queens were captured (two *B. cryptarum,* two *B. lucorum,* and one *B. magnus*), too few to draw conclusions about their phenology.

### Effects of weather on activity of the three species

**Figure 4:**
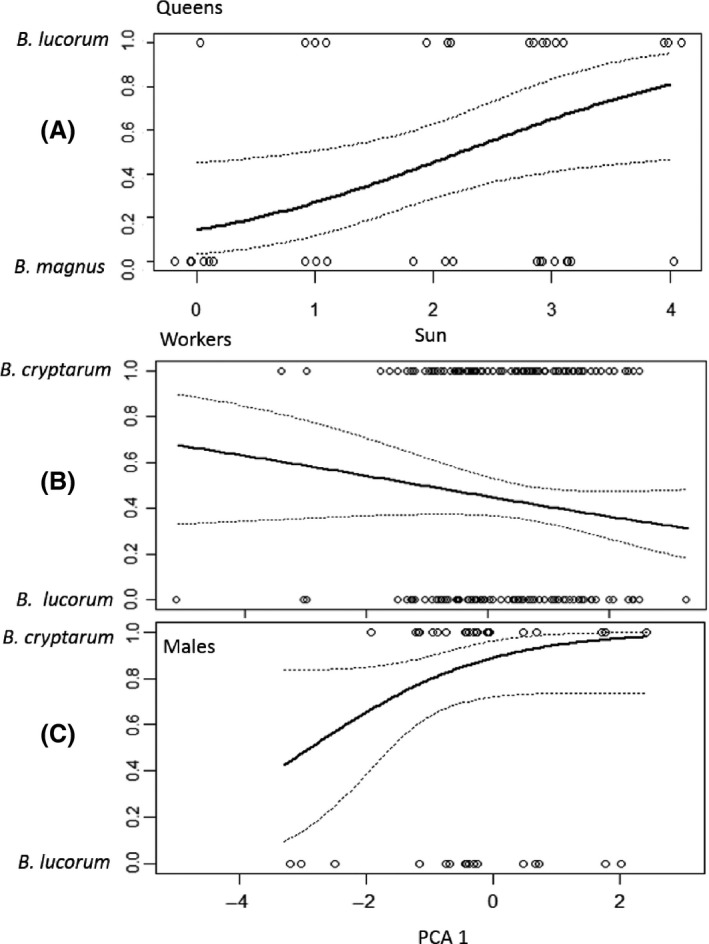
Differences in the effects of weather variables on the activity of *lucorum* complex species. (A) The level of sunshine differentially affected the abundance of queens of *B. lucorum* and *B. magnus*: The sun axis represents a scale ranging from 0 (heavy complete cloud cover) to 4 (<25% cloud cover; see Table S1). In (B & C), PCA 1 represents a scale where low values indicate cool cloudy conditions and higher values indicate warmer, sunnier conditions (see Fig. S4): Figures display the relative impact of changes in this weather axis on the activity of workers (B) and males (C) of *B. cryptarum* compared to *B. lucorum*. Trend lines are model fits from a generalised linear model with 95% confidence intervals.

We used PCA to reduce the number of weather variables. The first PCA axis, which accounted for 40.28% of the total variation, exhibited a positive correlation with temperature and amount of sunshine: Increasingly positive values represent generally warmer and sunnier conditions (Figure S4B). The three species showed variation along this axis: The average observations for *B. magnus* were lower (negative values) than for *B. lucorum* (positive values), observations for *B. cryptarum* were intermediate (Figure S4A). The second PCA axis was negatively associated with the wind speed; however, there was little variation between the species in this metric (Figure S4A). Therefore, we substituted values from the first PCA axis (PCA 1) for the explanatory variables “temperature” and “level of sunshine” in subsequent analyses for worker and males. For overwintered queens, we found that using PCA 1, which represents a combined measure of warmth and sunniness, instead of the separate sunshine and temperature variables did not improve the best model; therefore, we retained both weather variables. Controlling for phenological variation (by retaining date as a fixed effect explanatory variable), *B. magnus* queens were relatively less active than *B. lucorum* in sunny conditions and relatively more active when it was overcast (Fig. 4A & Table S3). We did not detect any significant effect of weather on the relative abundance of *B. cryptarum* queens in comparison with queens of either of the other species (Table S3).

Weather differentially affected worker activity for *B. cryptarum* and *B. lucorum*. Averaging across the whole season, *B. cryptarum* workers were relatively more common than *B. lucorum* workers when conditions were cooler and cloudier (although this effect was not significant: Fig. 4B & Table S5). Nevertheless*,* there was a significant effect of the interaction between the date quadratic term and PCA 1 (*χ* ^2^_2_ = 7.57, *P* = 0.02, Table S5). This interaction demonstrated that while *B. lucorum* workers were rare early and late in the season, becoming relatively more common around midsummer, the midsummer increase in relative abundance was more pronounced when it was warm and sunny, compared to cooler cloudy conditions (Figure S6). Weather did not significantly affect the relative abundance of *B. magnus* workers compared to either of the other species. Considering males, there were fewer *B. cryptarum* males encountered compared to *B. lucorum* in the coolest, cloudiest conditions (Fig. 4C & Table S7).

Temperature varies throughout the day, so we tested whether the differential temperature effects on the probability of activity in *B. cryptarum* and *B. lucorum* led to temporal separation of foraging across the day. Model selection using AICc incorporating time of day instead of PCA 1, favored models incorporating time (436.7 vs. 432.8 AICc points), but the pattern was the same. The significant interaction between the quadratic terms, date, and time of day (*χ* ^2^_4_ = 16.1, *P* < 0.005, Table S8) showed that the mid‐season peak in the relative abundance of *B. lucorum* workers was strongest early in the morning compared to later in the day. We did not detect differences in temporal activity in any other interspecific comparisons.

### Niche overlap between the bumblebee species

**Figure 5:**
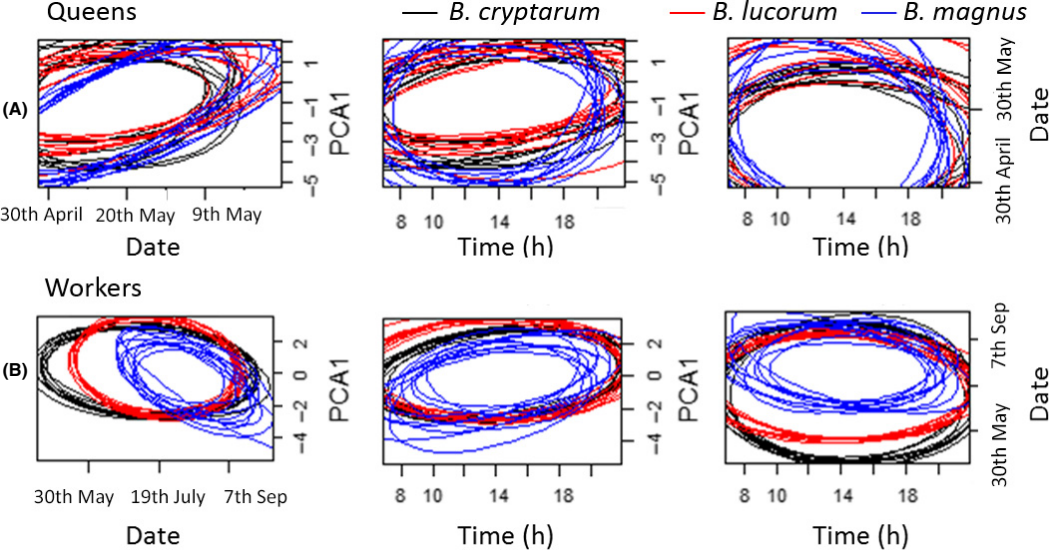
Comparisons of the niche size for the three *lucorum* complex species. Panels show ten random elliptical projections of niche region (N_R_) for each bumblebee species defined by pairs of variables for (A) queens and (B) workers. Niche regions were estimated based on the modeled influence of seasonal, weather and daily activity variables on occurrence. Each plot illustrates the projected niche region for a different combination of the three variables. PCA 1 represents a weather axis where low values indicate cool cloudy conditions and higher values indicate warmer, sunnier conditions (Fig. S4). Black lines represent *B. cryptarum*, red represent *B. lucorum* and blue represent *B. magnus*.

We defined the niche region (N_R_) exploited by each bumblebee species based on the date, weather conditions, and time of day when each individual was out foraging. Among queens, there was little difference in N_R_ and the probabilities of overlap were quite similar for all interspecific comparisons (overlap probability: 0.76–0.88; Fig. 5A & Table S10). Among workers, *B. magnus* had the smallest N_R_ (Fig. 5B), which is due in large part to a narrower range of dates (later in the season) when they were on the wing. *B. cryptarum* workers displayed the largest N_R,_ partly driven by the fact they were on the wing for the longest period (Fig. 5B). We therefore found strong asymmetry in the degree of interspecific niche overlap for workers. While *B. magnus* workers were highly likely to exploit the niche of *B. cryptarum* and *B. lucorum* workers (overlap probability: 0.94 and 0.95, respectively), the probability that *B. cryptarum* and *B. lucorum* workers were found in the niche of *B. magnus* workers was much lower (overlap probability: 0.47 and 0.50, respectively, Table S10).

### Forage use

Queens of all three bumblebee species were found feeding on a similar number of plant taxa (range 7–8). However, 52.7% of *B. magnus* queens (*n* = 14) were recorded visiting *Erica* spp., whereas only a single individual of both *B. cryptarum* (*n* = 13) and *B. lucorum* (*n* = 10) foraged on heather plants (Table S11). Previous studies (Waters et al. 2011; Scriven et al. 2015) suggest a tight association between *B. magnus* and heather (*C. vulgaris* and *Erica* spp.). We tested this explicitly and found that queens of the three bumblebee species differed significantly in their probability of foraging on heather, compared to all other plant species (*χ* ^2^_2_ = 6.79, *P* < 0.05); parameter estimates show this was due to *B. magnus* queens foraging on this taxon more often than *B. cryptarum*, but post hoc tests did not detect any individually significant differences between pairs of species (*P* > 0.05).

Concerning workers, the plants most commonly visited by *B. cryptarum* were *C. vulgaris* (17.1%) and *Erica* spp. (15.3%). However, the proportional representation of these species in the *B. cryptarum* diet was markedly lower than for *B. magnus*, of which 84.6% foraged on *C. vulgaris* (61.5%) or *Erica* spp. (23.1%, Table S12). Only 26% of *B. lucorum* workers were foraging on these plant taxa. We found that, like queens, workers of the three bumblebee species also differed significantly in their probability of foraging on heather compared to all other plant species (*χ* ^2^_2_ = 15.15, *P* < 0.001): *Erica* spp. and *C. vulgaris* made a significantly larger contribution to the diet of *B. magnus* workers than to the diets of *B. cryptarum* or *B. lucorum* (*P* < 0.01). This may in part be because *B. magnus* workers are only on the wing during the flowering period of *Erica* spp. and *C. vulgaris*; whereas workers of the other two species were also on the wing when these plant species were not available as food sources. However, considering just the period when *Erica* spp. and *C. vulgaris* were in flower, the bumblebee species still foraged on these plant species to differing extents (*χ* ^2^_2_ = 16.92, *P* < 0.001): Comparisons were individually significant for both *B. magnus* ‐ *B. cryptarum* (*P* < 0.05) and *B. magnus ‐ B. lucorum* (*P* < 0.005). Moreover, during this heather flowering period, the probability of *B. cryptarum* foraging on *Erica* spp. and *C. vulgaris* was significantly greater than for *B. lucorum* (*P* < 0.05). The forage plant that made the greatest contribution to the diet of *B. lucorum* workers was *Rubus* sp. (27.6%), a species that contributed significantly less (12.9%) to the diet of *B. cryptarum* (*χ* ^2^_1_ = 9.97, *P* = 0.002, Table S12).

Males of *B. cryptarum* and *B. lucorum* had a similar diet breadth; however, their diets only overlapped on four plant taxa (total taxa = 9 and 7, respectively, Table S13). The plant most frequently visited by *B. cryptarum* males (66.1%) was *Succisa pratensis*, which contributed significantly less (29.6%) to the diet of *B. lucorum* males (*χ* ^2^_1_ = 8.3, *P* = 0.003). The other most often utilised forage plant for *B. lucorum* males was *Chamerion angustifolium* (29.6%)*,* which was not used at all by *B. cryptarum* (Fisher's exact test *P* < 0.001). Only two *B. magnus* males were found preventing assessment of diet breadth for males of this species.

We calculated between‐species dissimilarity in diet composition using the Bray–Curtis coefficient (Bray and Curtis 1957). A Bray–Curtis distance value of zero indicates that the two forage assemblages are identical for both species, whereas a value of one means they are completely dissimilar. The degree of dietary dissimilarity between *B. cryptarum* and *B. lucorum* was almost identical for queens and workers (0.30 and 0.31, respectively), whereas for males, it was greater (0.69, Table 2). Among queens, the greatest difference in diet composition was between *B. cryptarum* and *B. magnus*. For workers, the degree of dissimilarity was greatest between *B. magnus* and the other two species (>0.6); the diets of *B. lucorum* and *B. cryptarum* workers were more similar (0.31, Table 2).

**Table 2:** Differences in the plant species assemblages used as forage resources by the three *lucorum* complex species. Bray–Curtis distance measures showing the dissimilarity between the diets of each caste of each of the three bumblebee species. A value of 0 indicates that the two assemblages were identical, whereas a value of 1 indicates that they were completely different. *B. magnus* males are not included because the sample size was too low: Only overwintered queens are included

	Caste	*B. cryptarum*	*B. lucorum*
*B. lucorum*	Queens	0.30	**–**
	Workers	0.31	–
	Males	0.69	–
*B. magnus*	Queens	0.51	0.35
	Workers	0.64	0.72

## Discussion

The sympatric occurrence of cryptic species challenges ecological theory because their strong biological similarity should generate intense interspecific competition and potential competitive exclusion (Gause 1932; Hardin 1960; Cothran et al. 2013; Van Campenhout et al. 2014). Nevertheless, the *lucorum* complex contains three cryptic bumblebee species with near‐identical morphology, which co‐occur across large parts of the UK (Bertsch et al. 2005; Waters et al. 2011; Carolan et al. 2012; Scriven et al. 2015) and elsewhere (Murray et al. 2008; Stanley et al. 2013; Williams et al. 2012b). In this study of *B. lucorum*,* B. cryptarum,* and *B. magnus,* we demonstrate clearly that although the niches of these three cryptic species overlap considerably, they do have distinct ecologies. We reveal niche utilisation differences that may be sufficient to prevent competitive exclusion by reducing the intensity of interspecific competition. We also provide the first reliable evidence for differences in their phenology.

We focussed on interspecific variation in three fundamental biotic and abiotic niche‐use dimensions at a single site: differences in responses to weather conditions, different forage use, and different temporal activity patterns. *B. magnus* had the most distinct niche. It has a narrow, highly specialised diet, feeding predominantly on species of heather plant (Ericacae). The phenology of all three *B. magnus* castes was delayed relative to the other two species; furthermore, queens of *B. magnus* were more active in overcast conditions, compared to those of *B. lucorum*. In contrast, although all castes of *B. cryptarum* and *B. lucorum* were on the wing for the same period of time, *B. lucorum* workers showed a strong peak in abundance around midsummer, followed by an earlier peak in the production of male reproductives. *B. lucorum* worker activity was skewed toward warmer, sunnier conditions compared to *B. cryptarum,* and the elevated abundance of *B. lucorum* in midsummer was strongest in warm conditions. *B. cryptarum* had a different phenology: Worker numbers increased faster, and decreased more slowly as the season progressed, compared to *B. lucorum*. This may in part be because *B. cryptarum* is better adapted for activity in colder conditions: its workers foraged more in cooler cloudier conditions than did *B. lucorum* (though, conversely, we captured fewer *B. cryptarum* males in cool cloudy conditions). There was some subtle diurnal temporal separation of foraging behavior between *B. lucorum* and *B. cryptarum*: In midsummer, when *B. lucorum* was relatively most common, its workers were more numerous early in the morning and least active in the afternoon.

The broad conclusions that *B. lucorum* is adapted for activity in warmer sunnier conditions, whereas *B. magnus* and *B. cryptarum* are adapted to forage in cooler cloudier conditions, recapitulates previous species distribution analysis. Scriven et al. (2015) showed that across Great Britain, *B. magnus* and *B. cryptarum* were more commonly found at sites with lower summer temperatures. Our current findings may help explain why *B. lucorum* is ecologically dominant throughout much of lowland southern and eastern England where it is warmer and sunnier, whereas *B. cryptarum* and *B. magnus* tend to occur in upland and northerly locations (Scriven et al. 2015). Similarly, our demonstration that the colony cycle of *B. magnus* is delayed in the season relative to the other two species is supported by previous work at our fieldwork location in August 2013, when both workers and males of *B. lucorum* and *B. cryptarum* were present, whereas only *B. magnus* workers were detected (Scriven et al. 2015). Such variation in the timing of male production in these three species may reduce the likelihood of hybridisation, thereby reinforcing reproductive isolation.

The observation that *B. magnus* preferentially chose to forage on heather plants supports observations that *B. magnus* is more commonly found on heather moorland (Scriven et al. 2015). Previous studies also revealed differences in the diet of these three species; however, these studies have combined data from multiple sites. For example, Scriven et al. (2015) found that across the UK, *B. lucorum* had the broadest diet and *B. magnus* had the narrowest diet breadth. However, it is not clear whether this resulted from different forage preferences, because bee species with restricted geographic ranges may have access to a restricted range of forage plants. In the present study, all the bumblebees had the same forage plants to choose from; despite this, clear differences in their diets remained.

Cryptic species provide important case studies to investigate the types of niche‐utilisation traits that diverge most readily after speciation events. The *lucorum* complex species seem to have diverged relatively recently (<100,000 years ago; based on COI divergence and diversity reported by Carolan et al. 2012 and Murray et al. 2008, using the approach of Jiggins and Tinsley 2005, and the standard insect molecular clock of Brower 1994) and previous work suggests that *B. magnus* and *B. cryptarum* are the most closely related of the three (Bertsch et al. 2005; Murray et al. 2008; Williams et al. 2012b). The interspecific niche differentiation we have observed may have underlain this speciation process. Evolution in metabolic pathways or morphology may be responsible for the thermal specialisation for activity in cooler conditions exhibited by *B. cryptarum* and *B. magnus*. Bumblebees are facultatively endothermic, requiring preflight metabolic warm‐up, large body size, and thoracic insulation for flight. Thermal specialisation is an important mechanism that may reduce the strength of interspecific competition; it may also mean that the members of a community of bee species can offer complementary pollination services to plants (Herrera 1997; Peat et al. 2005; Lye et al. 2010; Frund et al. 2013). However, in our dataset, air temperature and time of day covaried (after accounting for seasonal changes); therefore, it is not possible to definitively rule out divergent circadian rhythms as an explanation for interspecific differences in the association between activity and temperature. The most dramatic aspect of niche divergence within this cryptic species complex is the strong preference in *B. magnus* to forage on the heathers, *C. vulgaris, E. cinerea,* and *E. tetralix* to the exclusion of other potentially suitable species that were common in the area.

We have shown significant differences along three niche dimensions of three cryptic species that are likely to facilitate their coexistence. However, there is also considerable niche overlap, which must lead to competition. Direct interference competition between these bumblebee species is unlikely, but there is the potential for exploitative competition for resources. An important resource for which both inter‐ and intraspecific competition may occur is pollen and nectar. Therefore, as all three *lucorum* complex species are present at this site and draw on similar resources, the differences found in their use of forage plants may possibly be driven by competition and reflect differences in their realised niches, rather than fundamental niches. In contrast, the other niche dimensions investigated, phenology and response to weather, are less likely to be influenced by competition, and may thus represent interspecific differences in the fundamental niche. Patterns of bumblebee visitation to the same plant species can vary through space and time, potentially as a response to variation in pollen abundance and quality (Vaudo et al. 2014) and also to avoid interspecific competition (Lye et al. 2010). We found that interspecific niche overlap was higher for queens than it was for either workers or males. However, seasonal changes in the abundance of forage plants relative to bumblebees mean that it is hard to determine the impact of this shift in niche overlap on the strength of interspecific competition acting on the different castes. In terms of the temporal and weather niche, *B. magnus* workers were most differentiated, with worker production delayed compared to the other species, potentially in order to coincide with the flowering of their principal forage plants. Consequently, despite being most differentiated, the niche of *B. magnus* workers was situated mostly within the niche region of the other two species. This creates an asymmetry in niche overlap, with *B. magnus* potentially suffering more strongly from competition than either of the species it interacts with. However, more specialised species are presumed to be more efficient in their preferred conditions than generalists (Pianka 1994). In the UK, *C. vulgaris* forms an important, and often dominant, component of both upland and lowland heaths (Thompson et al. 1995; Groves et al. 2012). As a specialist on heather species, *B. magnus* may be an optimal forager on this resource, exploiting it more efficiently than the other species. Furthermore, by delaying worker production until *C. vulgaris* and *Erica* spp. flower, *B. magnus* could be able to profit from this extremely abundant resource, limiting the impact of overlap along the other niche dimensions, while avoiding worker competition with the other two bumblebee species earlier in the season when resources are more limited.

The niche differences that we have observed in this study may assist co‐occurrence of these cryptic species by causing variation in the responses of each species to spatio‐temporal heterogeneity in seasonally changing foraging sites. When the resources available for colony growth are continuously changing, the competitive relations between colonies of different species can be reversed, leading to the maintenance of a larger number of species in a region (Westphal et al. 2006). The composition and abundance of bee populations have been shown to undergo considerable variation between years (Minckley et al. 1999; Oertli et al. 2005; Iserbyt and Rasmont 2012). Iserbyt and Rasmont (2012) found that in one mountainous region, the dominant bumblebee species one year was seldom dominant another year, some species disappeared totally for several years and the proportion of permanent species was low. We observed clear abundance differences in the bumblebee species: *B. cryptarum* was the most common species and *B. magnus* was by far the least abundant. Yet previous sampling in 2011 found the most common species to be *B. magnus* (50.5%), whereas *B. cryptarum* was the least common (19.4%, Scriven et al. 2015). Clearly, the relative proportions of *B. magnus* and *B. cryptarum* can vary strongly between years, suggesting that the two species do not respond to environmental fluctuations in the same way. This could therefore represent a system where ecological divergence, niche partitioning, and spatio‐temporal heterogeneity in the environment mean that none of the three species is able to consistently exclude another to the point of local extinction. This has considerable implications for conservation, as small alterations to any of these dimensions could modify interspecific interactions putting one or more species at risk. *B. magnus* relies heavily on threatened and declining heathland habitat; further losses could therefore shift the balance and seriously affect populations of this species. Studying these species over several consecutive years may reveal trends in the population composition linked to annual climatic variations and allow us to understand in more detail what climatic factors affect the success of these three species. Similarly, broadening the study to include other sites would demonstrate whether these patterns are consistent across areas.

The discovery of co‐occurring cryptic species presents problems for several areas of ecological theory: The limits of ecological differentiation required for species coexistence, phylogenetic limiting similarity, and competitive exclusion (Violle et al. 2011; Gabaldón et al. 2013; Van Campenhout et al. 2014). We show that a combination of varying levels of ecological divergence in different niche dimensions and spatio‐temporal heterogeneity in the environment may contribute to the persistence of cryptic species in sympatry. Furthermore, our study suggests that cryptic species provide distinct and unique ecosystem services, clearly demonstrating that morphological similarity between species does not necessarily equate to ecological equivalence.

## Conflict of Interest

None declared.

## Data accessibility

Data available from the Dryad Digital Repository: doi:10.5061/dryad.js1gm.

## Supporting information


**Table S1**. Scale used for categorising amount of sun.
**Table S2**. Scale used for categorising rainfall.
**Table S3**. Phenological variation and differences in responses to weather conditions between over‐wintered queens of each bumblebee species.
**Figure S4.** Results of principal component analysis (PCA) on the variation in weather condition metrics (sun, wind speed, rain and air temperature) when each individual bumblebee was encountered. Axis 1 (PCA 1) and Axis 2 (PCA 2) describe 40.3% and 26.4% of the total variation respectively.
**Table S5.** Differences in phenology and responses to weather conditions of workers for each bumblebee species.
**Figure S6.** The effect of seasonality and changing weather conditions on the abundance of *B. cryptarum* and *B. lucorum* workers on the wing.
**Table S7.** Differences in phenology and responses to weather conditions between males of *B. cryptarum* and *B. lucorum*.
**Table S8.** Changes in seasonal and daily activity in workers of *B. cryptarum* and *B. lucorum*.
**Figure S9.** The effect of date and time of day on the abundance of *B. cryptarum* and *B. lucorum* workers on the wing.
**Table S10.** Differences in niche overlap between queens and workers of each of the three *lucorum* complex species.
**Table S11.** Forage use and measures of diet breadth for *lucorum* complex over‐wintered queens.
**Table S12.** Forage use and measures of diet breadth for *B. lucorum* complex workers.
**Table S13.** Forage use and measures of diet breadth for *B. lucorum* complex males.
