# Supplementary material for: Niche partitioning in a sympatric cryptic species complex
Source: Ecol Evol. 2016 Jan 28;6(5):1328–39. doi: 10.1002/ece3.1965 (PMC4730923; doi:10.1002/ece3.1965)
Supplement: Supplementary file 1 — Table S1. Scale used for categorising amount of sun. Table S2. Scale used for categorising rainfall. Table S3. Phenological variation and differences in responses to weather conditions between over‐wintered queens of each bumblebee species. Figure S4. Results of principal component analysis (PCA) on the variation in weather condition metrics (sun, wind speed, rain and air temperature) when each individual bumblebee was encountered. Axis 1 (PCA 1) and Axis 2 (PCA 2) describe 40.3% and 26.4% of the total variation respectively. Table S5. Differences in phenology and responses to weather conditions of workers for each bumblebee species. Figure S6. The effect of seasonality and changing weather conditions on the abundance of B. cryptarum and B. lucorum workers on the wing. Table S7. Differences in phenology and responses to weather conditions between males of B. cryptarum and B. lucorum. Table S8. Changes in seasonal and daily activity in workers of B. cryptarum and B. lucorum. Figure S9. The effect of date and time of day on the abundance of B. cryptarum and B. lucorum workers on the wing. Table S10. Differences in niche overlap between queens and workers of each of the three lucorum complex species. Table S11. Forage use and measures of diet breadth for lucorum complex over‐wintered queens. Table S12. Forage use and measures of diet breadth for B. lucorum complex workers. Table S13. Forage use and measures of diet breadth for B. lucorum complex males. [file ECE3-6-1328-s001.docx]

**Supporting Information**

**Table S1**. Scale used for categorising amount of sun.

| **Scale value** | **Description** |
| --- | --- |
| 0 | Heavy complete cloud cover |
| 1 | Light complete cloud cover |
| 2 | Some breaks in cloud, > 75% cloud cover |
| 3 | 25-75% cloud cover |
| 4 | < 25% cloud cover |

**Table S2**. Scale used for categorising rainfall.

| **Scale value** | **Description** |
| --- | --- |
| 0 | Completely dry, no precipitation |
| 1 | Light mist |
| 2 | Drizzle |
| 3 | Light rain |
| 4 | Rain |
| 5 | Heavy, torrential rain |

**Table S3**. Phenological variation and differences in responses to weather conditions between over-wintered queens of each bumblebee species. Results from pairwise analyses between the species, using generalised linear models with binary error distributions, testing the effects of date and the amount of sun on the relative abundance of over-wintered queens. Significant results are shown in italics. Values for the best model are shown in bold, other values are included for comparisons. Negative parameter estimates indicate a decrease in the probability of individuals belonging to the first species in the comparison whereas a positive estimate indicates an increase in the probability of individuals belonging to the first species.

|  | ***B. cryptarum - B. lucorum*** | | | |  | ***B. lucorum - B. magnus*** | | | |  | ***B. cryptarum - B. magnus*** | | | |
| --- | --- | --- | --- | --- | --- | --- | --- | --- | --- | --- | --- | --- | --- | --- |
| **Parameter** | **Estimate** | **SE** | **χ^2^** | **P** |  | **Estimate** | **SE** | **χ^2^** | **P** |  | **Estimate** | **SE** | **χ^2^** | **P** |
| Date | -0.01 | 0.03 | 0.10 | 0.75 |  | **-0.06** | **0.03** | 4.16 | *0.04* |  | **-0.05** | **0.03** | 4.17 | *0.04* |
| Sun | -0.22 | 0.31 | 0.52 | 0.47 |  | **0.81** | **0.36** | 6.19 | *0.01* |  | 0.45 | 0.29 | 2.66 | 0.10 |

**
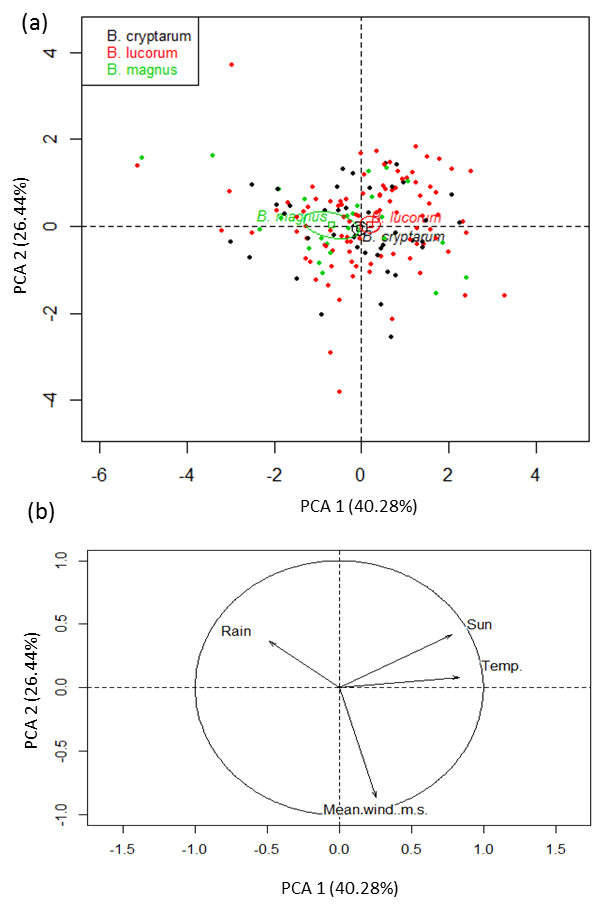
**

**Figure S4.** Results of principal component analysis (PCA) on the variation in weather condition metrics (sun, wind speed, rain and air temperature) when each individual bumblebee was encountered. Axis 1 (PCA 1) and Axis 2 (PCA 2) describe 40.3% and 26.4% of the total variation respectively. (a) Each point represents an individual bee. The square boxes show the average observations for each species and the ellipses show the 95% confidence levels around these values. (b) Vector plot showing the contribution of different weather variables to PCA 1 and 2.

**Table S5.** Differences in phenology and responses to weather conditions of workers for each bumblebee species. Results from pairwise analyses between the species, using generalised linear models with binary error distributions, testing the effects of date (with linear and quadratic terms) and PCA 1on the relative abundance of workers. PCA 1 represents a scale where low values indicate cool cloudy conditions and higher values indicate warmer, sunnier conditions (Fig. S4). Significant results are shown in italics. Values for the best model are shown in bold, other values are included for comparisons. Negative parameter estimates indicate a decrease in the probability of individuals belonging to the first species in the comparison whereas a positive estimate indicates an increase in the probability of individuals belonging to the first species.

|  | ***B. cryptarum - B. lucorum*** | | | |  | ***B. lucorum - B. magnus*** | | | |  | ***B. cryptarum - B. magnus*** | | | |
| --- | --- | --- | --- | --- | --- | --- | --- | --- | --- | --- | --- | --- | --- | --- |
| **Parameter** | **Estimate** | **SE** | **χ^2^** | **P** |  | **Estimate** | **SE** | **χ^2^** | **P** |  | **Estimate** | **SE** | **χ^2^** | **P** |
| Date | **-6.80** | **2.40** |  |  |  | **-0.04** | **0.01** | 9.90 | *0.002* |  | **-29.90** | **13.47** |  |  |
| Date^2^ | **9.56** | **2.68** | 14.20 | *< 0.001* |  | 11.89 | 8.03 | 3.29 | 0.07 |  | **13.44** | **8.48** | 4.14 | *0.04* |
| PCA 1 | **-0.11** | **0.10** | 0.63 | 0.429 |  | -0.86 | 0.87 | 0.45 | 0.50 |  | -0.94 | 1.27 | 0.05 | 0.82 |
| Date: PCA 1 | **-3.66** | **2.26** |  |  |  | 16.72 | 11.76 |  |  |  | 16.48 | 18.45 |  |  |
| Date^2^: PCA 1 | **1.73** | **1.71** | 7.57 | *0.02* |  | -15.43 | 11.80 | 2.41 | 0.30 |  | -10.15 | 11.08 | 0.99 | 0.61 |


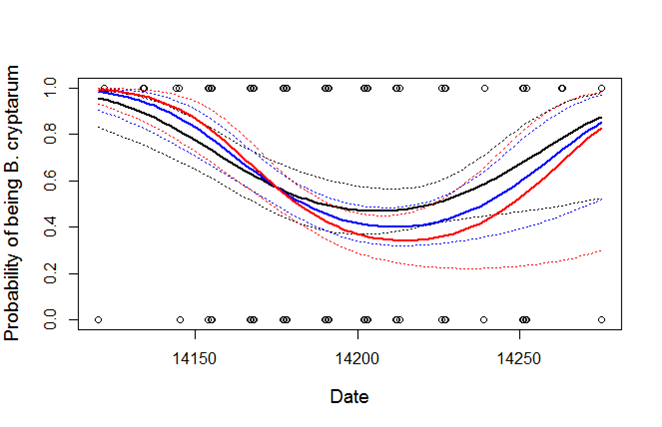


*B. cryptarum*

*B. lucorum*

30^th^ May

19^th^ July

7^th^ September

**Figure S6.** The effect of seasonality and changing weather conditions on the abundance of *B. cryptarum* and *B. lucorum* workers on the wing. Trend lines are model fits from generalised linear models with 95% confidence intervals. Black lines represent a low PCA 1 score (1^st^ quartile) hence cool, cloudy conditions; blue lines represent a mid value for PCA 1 (median) and red lines indicate a high PCA 1 score (3^rd^ quartile) hence warm, sunny conditions (see Fig. S4 for details of PCA 1).

**Table S7.** Differences in phenology and responses to weather conditions between males of *B. cryptarum* and *B. lucorum.* Results from pairwise analyses between the species, using generalised linear models with binary error distributions, testing the effects of date (with linear and quadratic terms) and PCA 1on the relative abundance of males. PCA 1 represents a scale where low values indicate cool cloudy conditions and higher values indicate warmer, sunnier conditions (Fig. S4). Significant results are shown in italics. Negative parameter estimates indicate a decrease in the probability of individuals belonging to the first species in the comparison whereas a positive estimate indicates an increase in the probability of individuals belonging to the first species. *B. magnus* males were not included because the sample size was very low.

|  | ***B. cryptarum - B. lucorum*** | | | |
| --- | --- | --- | --- | --- |
| **Parameter** | **Estimate** | **SE** | **χ^2^** | **Prob > χ^2^** |
| Date | **9.69** | **2.56** |  |  |
| Date^2^ | **-7.83** | **2.98** | 4.31 | *0.04* |
| PCA 1 | **0.72** | **0.40** | 4.15 | *0.04* |

**Table S8 .** Changes in seasonal and daily activity in workers of *B. cryptarum* and *B. lucorum*. Results from pairwise analyses between the species, using generalised linear models with binary error distributions, testing the effects of date and time of day (with linear and quadratic terms) on the relative abundance of workers. Significant results are shown in italics. Negative parameter estimates indicate a decrease in the probability of individuals belonging to the first species in the comparison whereas a positive estimate indicates an increase in the probability of individuals belonging to the first species.

|  | ***B. cryptarum - B. lucorum*** | | | |
| --- | --- | --- | --- | --- |
| **Parameter** | **Estimate** | **SE** | **χ^2^** | **P** |
| Intercept | 0.26 | 0.14 |  |  |
| Date | -2.02 | 2.76 |  |  |
| Date^2^ | 13.27 | 3.58 | 17.86 | *<0.001* |
| Time | 3.07 | 2.69 |  |  |
| Time^2^ | 0.75 | 3.25 | 2.21 | 0.33 |
| Date x Time | -24.27 | 55.37 |  |  |
| Date^2^ x Time | -27.79 | 67.55 |  |  |
| Date x Time^2^ | -12.53 | 64.81 |  |  |
| Date^2^ x Time^2^ | 274.19 | 89.31 | 16.12 | *0.003* |

**
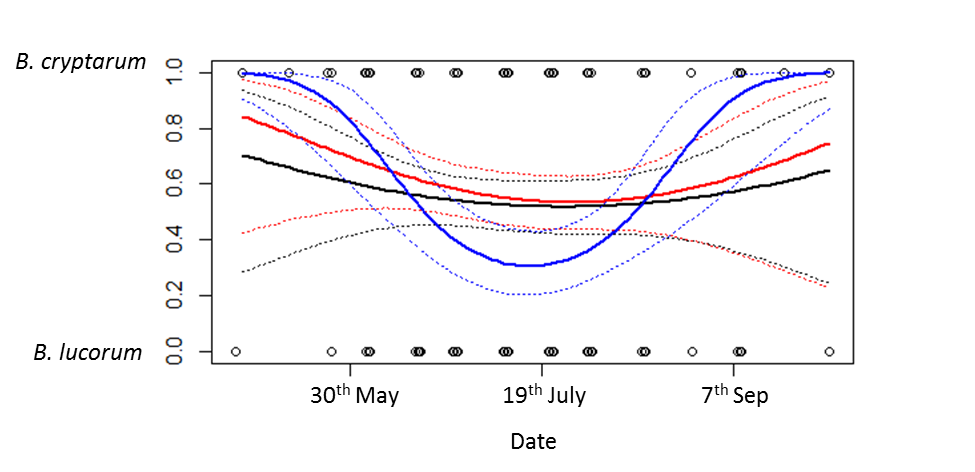
**

**Figure S9.** The effect of date and time of day on the abundance of *B. cryptarum* and *B. lucorum* workers on the wing. Trend lines are model fits from generalised linear models with 95% confidence intervals. Blue lines represent early in the day (10am); black lines represent the middle of the day (1pm) and red lines represent later in the day (4pm).

**Table S10.** Differences in niche overlap between queens and workers of each of the three *lucorum* complex species**.** Mean overlap probability between each bumblebee species at the niche region size α = 0.95 with 95% credible intervals. α represents the probability of an individual being found in the estimated niche region (Swanson, Lysy, & Power 2015). The niche regions were defined based on the date, weather and time of day when individuals were active. The overlap probability represents the likelihood that an individual from Species A will be found in the niche of Species B.

|  |  | Species B | | | | | | |
| --- | --- | --- | --- | --- | --- | --- | --- | --- |
|  |  | **Queens** | | |  | **Workers** | | |
| Species A | Parameter | ***B. cryptarum*** | ***B. lucorum*** | ***B. magnus*** |  | ***B. cryptarum*** | ***B. lucorum*** | ***B. magnus*** |
| ***B.*** | **Mean** | **-** | **0.88** | **0.78** |  | **-** | **0.90** | **0.47** |
| ***cryptarum*** | 2.5% | - | 0.71 | 0.56 |  | - | 0.84 | 0.29 |
|  | 97.5% | - | 0.98 | 0.94 |  | - | 0.95 | 0.67 |
| ***B. lucorum*** | **Mean** | **0.82** | **-** | **0.76** |  | **0.91** | **-** | **0.50** |
|  | 2.5% | 0.62 | - | 0.54 |  | 0.86 | - | 0.31 |
|  | 97.5% | 0.96 | - | 0.94 |  | 0.96 | - | 0.71 |
| ***B. magnus*** | **Mean** | **0.76** | **0.81** | **-** |  | **0.94** | **0.93** | **-** |
|  | 2.5% | 0.56 | 0.61 | - |  | 0.81 | 0.78 | - |
|  | 97.5% | 0.93 | 0.96 | - |  | 1.00 | 0.99 | - |

**Table S11.** Forage use and measures of diet breadth for *lucorum* complex over-wintered queens. Values represent the number of individuals of each bee species and, in parentheses, the percentage of the total number individuals of the corresponding bee species. Diet breadth is measured via rarefaction to estimate the number of plant species each bee species would be expected to visit in a total of 5 flower visits. Garden plant 1 was not a native wildflower, found in a garden that was not identified.

|  | ***B. cryptarum*** | ***B. lucorum*** | ***B. magnus*** | ***Total*** |
| --- | --- | --- | --- | --- |
| *Acer pseudoplatanus* | 2 (15.4) | 3 (30) |  | 5 |
| *Erica spp.* | 1 (7.7) | 1 (10) | 6 (42.9) | 8 |
| *Cotoneaster horizontalis* | 2 (15.4) | 1 (10) | 1 (7.1) | 4 |
| *Cytisus scoparius* | 2 (15.4) | 1 (10) | 2 (14.3) | 5 |
| *Erica cinerea* | 1 (7.7) | 2 (20) | 2 (14.3) | 5 |
| *Garden plant 1* | 1 (7.7) |  |  | 1 |
| *Lotus corniculatus* |  | 1 (10) | 1 (7.1) | 2 |
| *Rhododendron spp.* |  | 1 (10) | 1 (7.1) | 2 |
| *Salix spp.* | 3 (23.1) |  |  | 3 |
| *Taraxacum spp.* | 1 (7.7) |  |  | 1 |
| *Thymus polytrichus* |  |  | 1 (7.1) | 1 |
| **Total sample size** | **13** | **10** | **14** | **37** |
| **No. of plant taxa visited** | **8** | **7** | **7** | **11** |
| **Diet breadth (± S.D.)** | **4.3 ± 0.65** | **4.2 ± 0.68** | **3.6 ± 0.83** |  |

**Table S12.** Forage use and measures of diet breadth for *B. lucorum* complex workers. Values represent the number of individuals of each bee species and, in parentheses, the percentage of the total number individuals of the corresponding bee species. Diet breadth is measured via rarefaction to estimate the number of plant species each bee species would be expected to visit in a total of 10 flower visits. Garden plants 2-4 were exotic taxa found in gardens and not identified.

|  | ***B. cryptarum*** | ***B. lucorum*** | ***B. magnus*** | ***Total*** |
| --- | --- | --- | --- | --- |
| *Papaveroideae spp.* | 1 (0.6) |  |  | 1 |
| *Acer pseudoplatanus* | 4 (2.4) |  |  | 4 |
| *Aegopodium podagraria* | 8 (4.7) | 3 (2) |  | 11 |
| *Calluna vulgaris* | 29 (17.1) | 22 (14.5) | 8 (61.5) | 59 |
| *Centaura nigra* | 2 (1.2) | 6 (3.9) |  | 8 |
| *Chamerion angustifolium* | 13 (7.6) | 19 (12.5) |  | 32 |
| *Cirsium arvense* |  | 4 (2.6) |  | 4 |
| *Cotoneaster horizontalis* | 2 (1.2) |  |  | 2 |
| *Cytisus scoparius* | 5 (2.9) | 1 (0.7) |  | 6 |
| *Erica cinerea* | 26 (15.3) | 18 (11.8) | 3 (23.1) | 47 |
| *Erica tetralix* | 1 (0.6) |  |  | 1 |
| *Filipendula ulmaria* | 2 (1.2) |  |  | 2 |
| *Garden plant 2* | 1 (0.6) |  |  | 1 |
| *Garden plant 3* |  | 1 (0.7) |  | 1 |
| *Garden plant 4* |  | 1 (0.7) |  | 1 |
| *Hydrangea spp.* | 6 (3.5) | 3 (2) |  | 9 |
| *Lavandula spp.* |  | 1 (0.7) |  | 1 |
| *Lotus corniculatus* | 13 (7.6) | 4 (2.6) |  | 17 |
| *Lotus pedunculatus* | 4 (2.4) | 6 (3.9) |  | 10 |
| *Lupinus* | 1 (0.6) |  |  | 1 |
| *Narthecium ossifragum* | 1 (0.6) |  |  | 1 |
| *Potenilla erecta* | 3 (1.8) |  |  | 3 |
| *Rhododendron spp.* |  | 2 (1.3) |  | 2 |
| *Rubus spp.* | 22 (12.9) | 42 (27.6) |  | 64 |
| *Salix spp.* | 1 (0.6) |  |  | 1 |
| *Sanguisorba spp.* | 1 (0.6) |  |  | 1 |
| *Succisa pratensis* | 2 (1.2) | 2 (1.3) | 1 (7.7) | 5 |
| *Thymus polytrichus* | 4 (2.4) |  | 1 (7.7) | 5 |
| *Trifolium repens* | 17 (10) | 17 (11.2) |  | 34 |
| *Ulex europaeus* | 1 (0.6) |  |  | 1 |
| **Total sample size** | **170** | **152** | **13** | **335** |
| **No. of plant taxa visited** | **25** | **17** | **4** | **30** |
| **Diet breadth (± S.D.)** | **6.9 ± 1.2** | **6 ± 1.1** | **3.5 ± 0.6** |  |

**Table S13.** Forage use and measures of diet breadth for *B. lucorum* complex males. Values represent the number of individuals of each bee species and, in parentheses, the percentage of the total number individuals of the corresponding bee species. Diet breadth is measured via rarefaction to estimate the number of plant species each bee species would be expected to visit in a total of 25 flower visits. Diet breadth was not calculated for *B. magnus* as the sample size was too low.

|  | ***B. cryptarum*** | ***B. lucorum*** | ***B. magnus*** | **Total** |
| --- | --- | --- | --- | --- |
| *Calluna vulgaris* | 4 (7.1) | 4 (14.8) |  | 8 |
| *Centaura nigra* | 1 (1.8) | 1 (3.7) |  | 2 |
| *Chamerion angustifolium* |  | 8 (29.6) |  | 8 |
| *Erica cinerea* | 3 (5.4) |  | 1 (50) | 4 |
| *Hylotelephium telephium* | 6 (10.7) |  |  | 6 |
| *Lavandula spp.* | 2 (3.6) |  |  | 2 |
| *Lotus pedunculatus* | 1 (1.8) |  |  | 1 |
| *Nepeta racemosa* | 1 (1.8) |  |  | 1 |
| *Rubus spp.* |  | 2 (7.4) |  | 2 |
| *Senecio jacobaea* |  | 1 (3.7) |  | 1 |
| *Succisa pratensis* | 37 (66.1) | 8 (29.6) |  | 45 |
| *Symparicarpos albus* | 1 (1.8) | 3 (11.1) | 1 (50) | 5 |
| **Total sample size** | **56** | **27** | **2** | **85** |
| **No. of plant taxa visited** | **9** | **7** | **2** | **12** |
| **Diet breadth (± S.D.)** | **6.2 ± 1.1** | **6.8 ± 0.4** |  |  |

**References**

Swanson, H., Lysy, M. & Power, M. (2015). A new probabilistic method for quantifying n-dimensional ecological niches and niche overlap. *Ecology*, 96, 318–324.
